# Supplementary material for: Genome-wide analysis of genetic diversity and artificial selection in Large White pigs in Russia
Source: PeerJ. 2021 Jul 2;9:e11595. doi: 10.7717/peerj.11595 (PMC8256806; doi:10.7717/peerj.11595)
Supplement: Supplemental Information 1 [file peerj-09-11595-s001.docx]

| 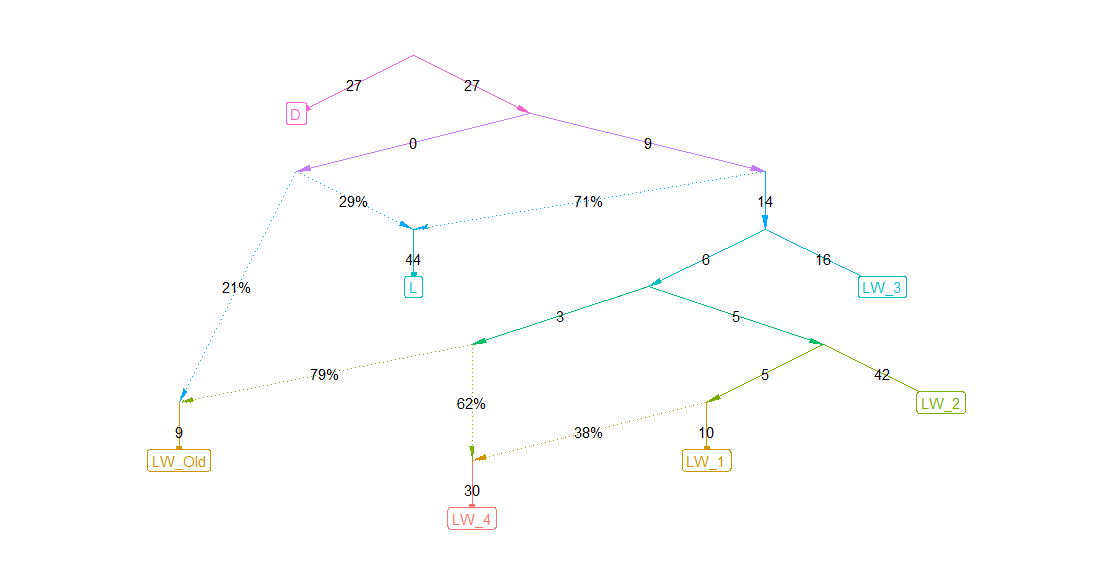 | 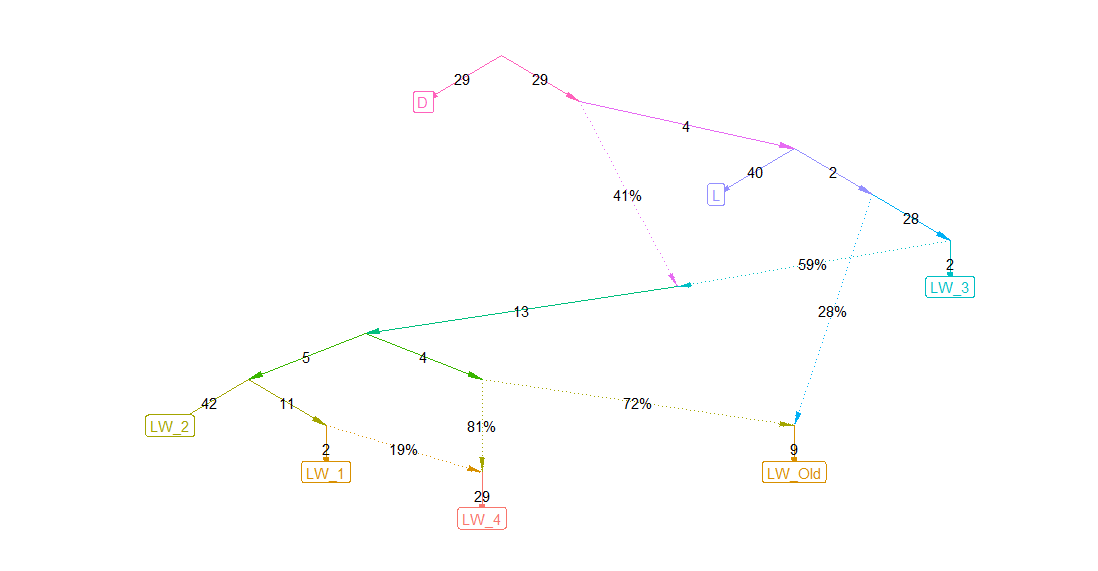 | 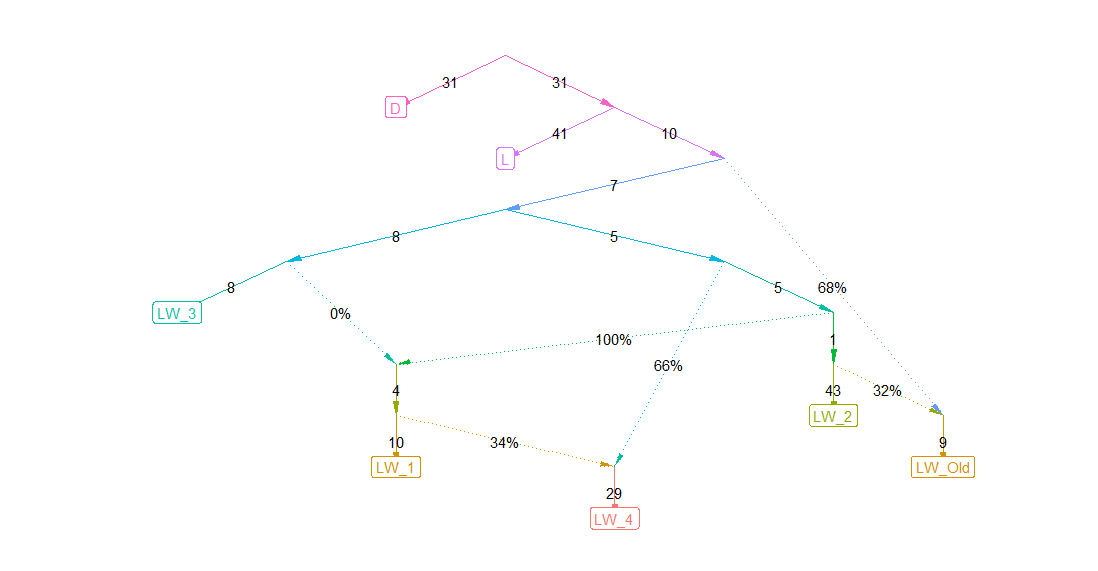 |
| --- | --- | --- |
| 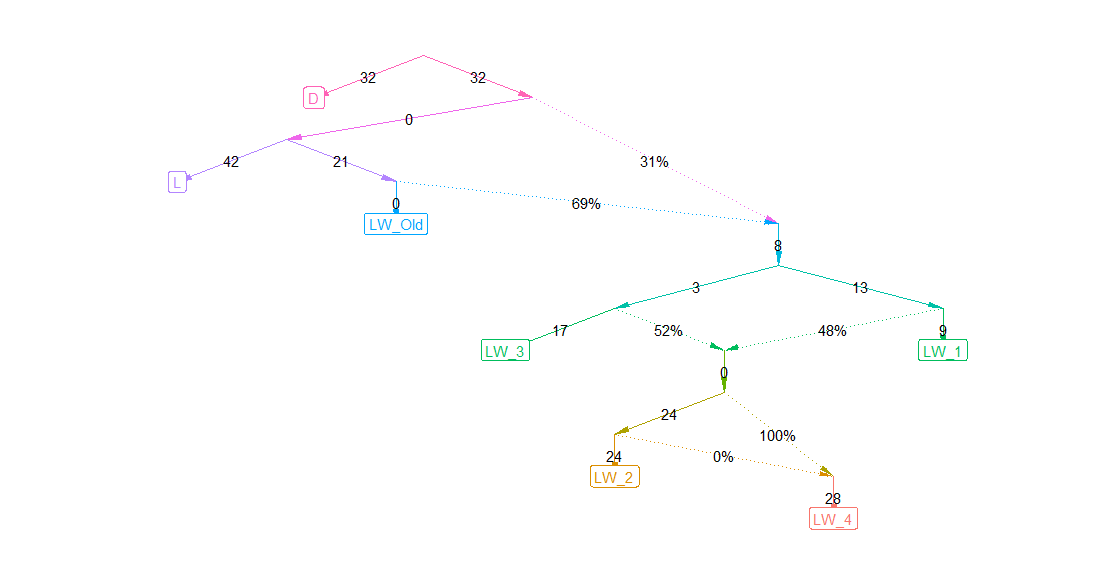 | 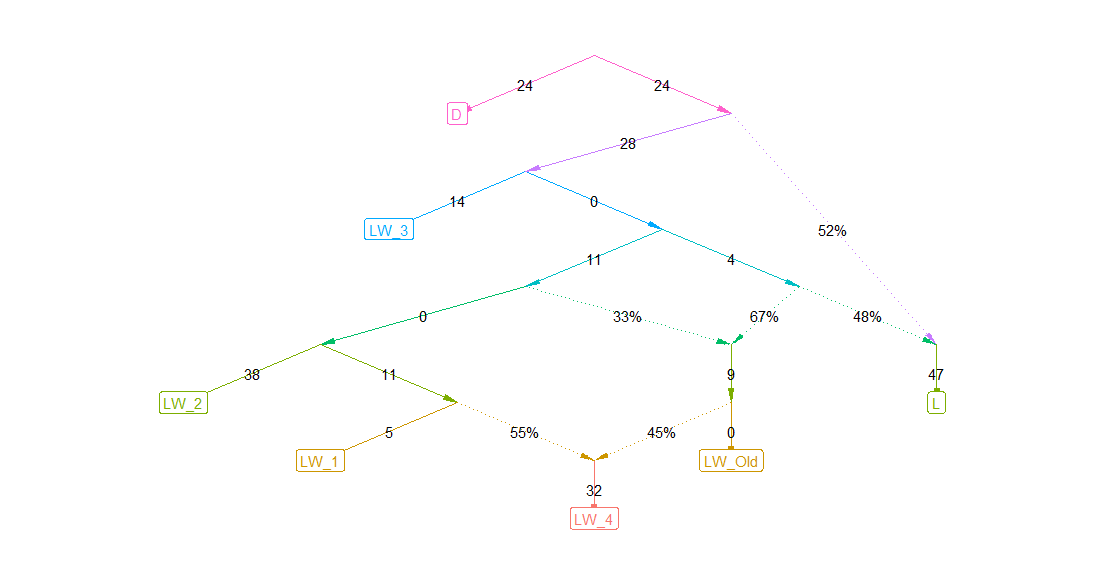 | 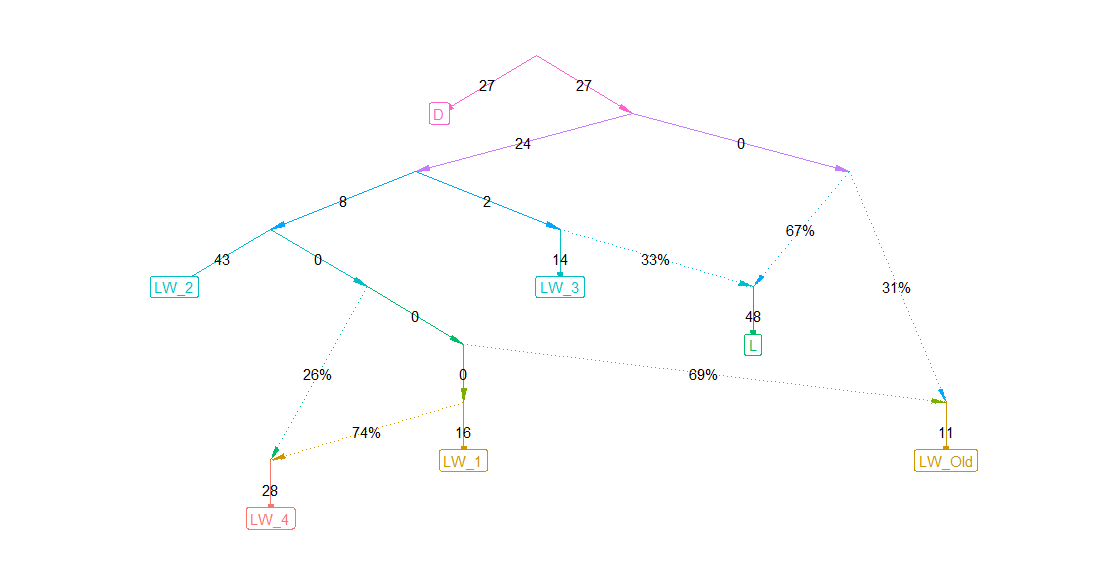 |
| 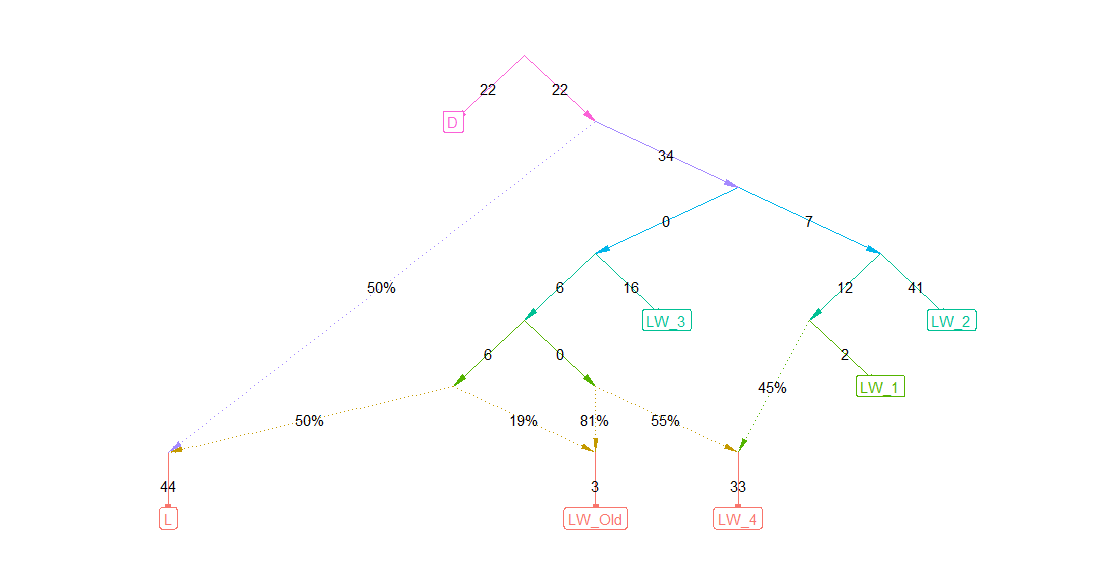 | 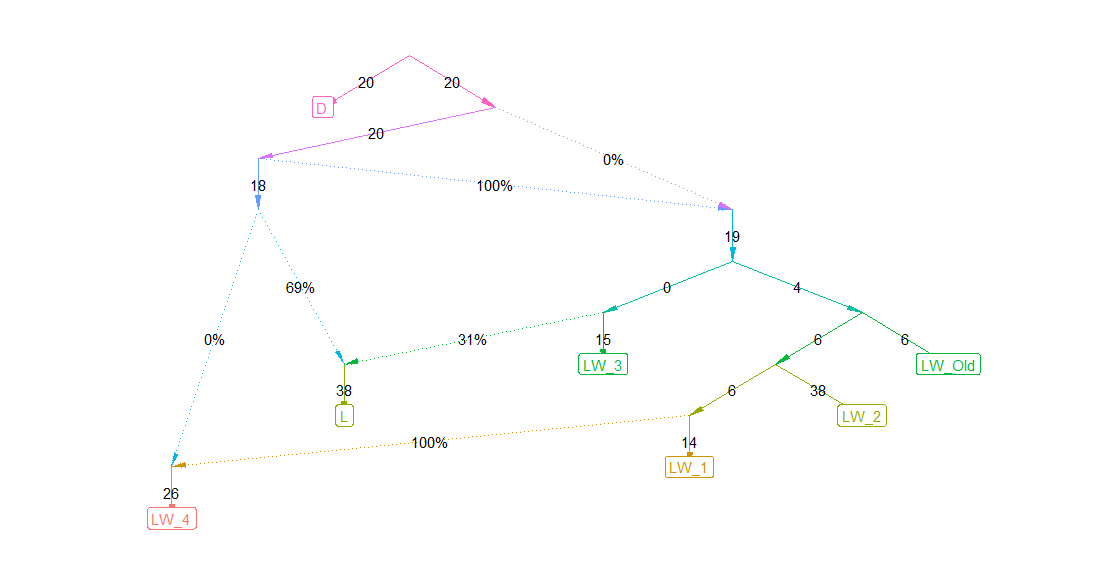 | 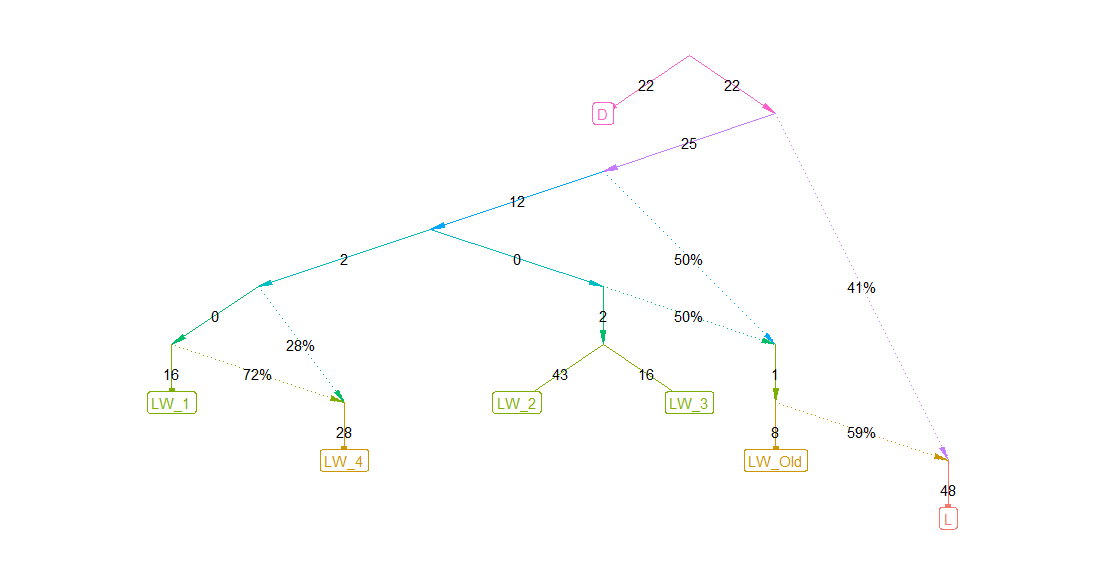 |

Figure S1. 9 best trees

Supp. Table 1. SNPs with the most significant signals, determined by the smoothed FST method

(LW_Old vs LW_New)

| Uploaded variation | SSC | Location | Gene | SNP rs |
| --- | --- | --- | --- | --- |
| ASGA0002802 | 1 | 51753405 | *-* | rs80857762 |
| ASGA0002804 | 1 | 51818265 | *RIMS1* | rs80800830 |
| H3GA0001710 | 1 | 51880036 | *RIMS1* | rs80913537 |
| ASGA0002809 | 1 | 51918328 | *RIMS1* | rs81354131 |
| ALGA0026233 | 4 | 81493481 | *-* | rs80848071 |
| MARC0083264 | 4 | 81652253 | *NME7* | rs81266074 |
| DIAS0004524 | 4 | 81769725 | *NME7* | rs334130525 |
| DRGA0004942 | 4 | 81916944 | *ATP1B1* | rs81302159 |
| DRGA0004943 | 4 | 81950375 | *ATP1B1* | rs81302085 |
| ALGA0026235 | 4 | 81986750 | *ATP1B1* | rs80865817 |
| ALGA0026236 | 4 | 82037515 | *-* | rs81001788 |
| MARC0091395 | 4 | 82167212 | *-* | rs81271948 |
| ALGA0026241 | 4 | 82242617 | *-* | rs81382225 |
| WU_10.2_4_89965423 | 4 | 82283682 | *DPT* | rs325785650 |
| INRA0015251 | 4 | 82416344 | *-* | rs339256957 |
| ALGA0026246 | 4 | 82530260 | *-* | rs80851024 |
| MARC0040853 | 4 | 82640649 | *-* | rs81232222 |
| ALGA0026251 | 4 | 82661799 | *TBX19* | rs80805879 |
| WU_10.2_4_90543975 | 4 | 82870646 | *GPR161* | rs319908869 |
| WU_10.2_4_90706777 | 4 | 83030262 | *DCAF6* | rs323730640 |
| WU_10.2_4_90870654 | 4 | 83194885 | *ADCY10* | rs80963344 |
| ASGA0020501 | 4 | 83256996 | *MPZL1* | rs80854124 |
| ALGA0026261 | 4 | 83269208 | *MPZL1* | rs80995413 |
| ALGA0026265 | 4 | 83331520 | *RCSD1* | rs80864601 |
| ALGA0026267 | 4 | 83351551 | *RCSD1* | rs80957430 |
| M1GA0006028 | 4 | 83366667 | *RCSD1* | rs80784543 |
| WU_10.2_4_91077081 | 4 | 83401317 | *RCSD1* | rs336604936 |
| H3GA0013230 | 4 | 83466133 | *CREG1* | rs80975099 |
| WU_10.2_4_91195648 | 4 | 83519869 | *CD247* | rs342976952 |
| MARC0059073 | 4 | 83575392 | *CD247* | rs80914031 |
| WU_10.2_4_91402132 | 4 | 83726358 | *POU2F1* | rs333593019 |
| ASGA0020515 | 4 | 83900192 | *STYXL2* | rs80966474 |
| ALGA0026283 | 4 | 83928709 | *GPA33* | rs80782001 |
| WU_10.2_4_139244621 | 4 | 127146360 | *-* | rs329145622 |
| ASGA0023314 | 4 | 127162411 | *U2* | rs81380889 |
| INRA0017729 | 4 | 127329631 | *-* | rs332269114 |
| ASGA0023322 | 4 | 127373937 | *-* | rs80969221 |
| MARC0056249 | 4 | 127417800 | *GBP2* | rs80908814 |
| WUR10000125_2 | 4 | 127441677 | *GBP2* | rs80800372 |
| WUR10000125 | 4 | 127441677 | *GBP2* | rs80800372 |
| ALGA0029524 | 4 | 127469240 | *KYAT3* | rs80931093 |
| H3GA0014860 | 4 | 127492397 | *KYAT3* | rs80868308 |
| ASGA0023344 | 4 | 127547467 | *GTF2B* | rs80972052 |
| ALGA0029534 | 4 | 127598486 | *PKN2* | rs80969690 |
| ASGA0023349 | 4 | 127650044 | *PKN2* | rs80881545 |
| ALGA0029538 | 4 | 127719021 | *-* | rs80974336 |
| ASGA0023354 | 4 | 127749186 | *-* | rs80908469 |
| MARC0045227 | 4 | 127806086 | *-* | rs80904492 |
| ASGA0023369 | 4 | 127831784 | *-* | rs80818844 |
| WU_10.2_4_140106958 | 4 | 127884005 | *-* | rs80961883 |
| WU_10.2_4_140281792 | 4 | 128105378 | *U6* | rs325421395 |
| MARC0071762 | 4 | 128112246 | *-* | rs80838629 |
| H3GA0014885 | 4 | 128161626 | *-* | rs80913794 |
| ALGA0029569 | 4 | 128175676 | *-* | rs80852519 |
| ASGA0023397 | 4 | 128203397 | *-* | rs80835488 |
| MARC0040196 | 4 | 128244327 | *-* | rs80819854 |
| WU_10.2_5_74089491 | 5 | 71470061 | *SLC2A13* | rs331576961 |
| ALGA0032799 | 5 | 71494266 | *SLC2A13* | rs81385262 |
| MARC0029526 | 5 | 71527091 | *SLC2A13* | rs81225104 |
| ALGA0032806 | 5 | 71555828 | *SLC2A13* | rs81385275 |
| ASGA0026259 | 5 | 71599622 | *SLC2A13* | rs81385292 |
| WU_10.2_5_74315950 | 5 | 71643337 | *SLC2A13* | rs340407698 |
| MARC0015072 | 5 | 71646843 | *SLC2A13* | rs81284021 |
| ASGA0026261 | 5 | 71672068 | *SLC2A13* | rs81385297 |
| M1GA0008010 | 5 | 71694586 | *-* | rs81385298 |
| WU_10.2_5_74398578 | 5 | 71725752 | *-* | rs337018445 |
| MARC0076572 | 5 | 71743106 | *-* | rs81261794 |
| ALGA0032819 | 5 | 71760723 | *-* | rs81385308 |
| MARC0051983 | 5 | 71843440 | *LRRK2* | rs81242782 |
| WU_10.2_5_74590144 | 5 | 71881066 | *LRRK2* | rs324990338 |
| DIAS0000357 | 5 | 71904955 | *LRRK2* | rs334486591 |
| WU_10.2_5_74693783 | 5 | 71984960 | *-* | rs346164486 |
| WU_10.2_5_75022148 | 5 | 72169605 | *CNTN1* | rs327321401 |
| MARC0033463 | 5 | 72192052 | *CNTN1* | rs81228490 |
| WU_10.2_5_75132228 | 5 | 72238893 | *CNTN1* | rs334635547 |
| DRGA0006005 | 5 | 72277629 | *CNTN1* | rs81302978 |
| ASGA0026270 | 5 | 72342967 | *CNTN1* | rs81385324 |
| ALGA0032834 | 5 | 72354872 | *CNTN1* | rs81385330 |
| INRA0019954 | 5 | 72393834 | *CNTN1* | rs331728472 |
| WU_10.2_5_75315384 | 5 | 72421553 | *CNTN1* | rs332833778 |
| WU_10.2_5_75349235 | 5 | 72455402 | *CNTN1* | rs196955486 |
| WU_10.2_5_75465827 | 5 | 72523648 | *-* | rs327502834 |
| MARC0083381 | 5 | 72561769 | *-* | rs80911861 |
| H3GA0016828 | 5 | 72610454 | *PDZRN4* | rs80795265 |
| INRA0019965 | 5 | 72637304 | *PDZRN4* | rs319495888 |
| WU_10.2_5_75602977 | 5 | 72660787 | *PDZRN4* | rs327536991 |
| MARC0056159 | 5 | 72681499 | *PDZRN4* | rs80790387 |
| WU_10.2_5_75652966 | 5 | 72710914 | *PDZRN4* | rs343745666 |
| WU_10.2_5_75678046 | 5 | 72735998 | *PDZRN4* | rs322114517 |
| ALGA0032852 | 5 | 72761996 | *PDZRN4* | rs80845858 |
| ALGA0032850 | 5 | 72774634 | *PDZRN4* | rs80792002 |
| MARC0022217 | 5 | 72783062 | *PDZRN4* | rs80953364 |
| ALGA0118476 | 6 | 45595002 | *ZNF146* | rs81325388 |
| ALGA0035270 | 6 | 45671421 | *-* | rs81395814 |
| ALGA0035271 | 6 | 45683888 | *-* | rs81395817 |
| ALGA0035274 | 6 | 45744000 | *ZNF461* | rs81395824 |
| MARC0013086 | 6 | 45804490 | *ZNF382* | rs81268225 |
| ASGA0028172 | 6 | 45823720 | *ZNF461* | rs81395834 |
| ASGA0083418 | 6 | 45854092 | *ZNF260* | rs80859145 |
| WU_10.2_6_99153897 | 6 | 106647339 | *GREB1L* | rs324979410 |
| WU_10.2_6_99269686 | 6 | 106823884 | *-* | rs332543333 |
| WU_10.2_6_99706201 | 6 | 107231177 | *-* | rs333865824 |
| WU_10.2_6_99964369 | 6 | 107499283 | *-* | rs342019377 |
| ASGA0084790 | 6 | 107577819 | *-* | rs81345398 |
| ALGA0113175 | 6 | 107604059 | *-* | rs81341898 |
| ASGA0090610 | 6 | 107705690 | *-* | rs81475846 |
| MARC0020347 | 6 | 107739202 | *-* | rs81289650 |
| ASGA0098402 | 6 | 107803551 | *-* | rs81318629 |
| ALGA0036285 | 6 | 108061282 | *-* | rs81390558 |
| ASGA0101922 | 6 | 114921710 | *DSC2* | rs81323111 |
| DRGA0006708 | 6 | 115063339 | *DSC1* | rs81299113 |
| H3GA0018683 | 6 | 115106736 | *-* | rs81390916 |
| MARC0043833 | 6 | 115272451 | *DSG1* | rs81235846 |
| ASGA0090954 | 6 | 115315450 | *DSG4* | rs81475861 |
| ASGA0098807 | 6 | 115348051 | *DSG3* | rs81477249 |
| MARC0096933 | 6 | 115491651 | *TTR* | rs81276080 |
| ASGA0089317 | 6 | 115501657 | *TTR* | rs81475807 |
| DIAS0004318 | 6 | 115503853 | *TTR* | rs55618548 |
| MARC0055381 | 6 | 115511066 | *B4GALT6* | rs81245564 |
| WU_10.2_6_108284251 | 6 | 115630713 | *-* | rs329207516 |
| DRGA0006714 | 6 | 115826701 | *-* | rs80852301 |
| INRA0022047 | 6 | 115933412 | *RNF138* | rs346414970 |
| ASGA0101965 | 6 | 115989400 | *-* | rs81323160 |
| MARC0050502 | 6 | 116094808 | *-* | rs81240150 |
| ALGA0036440 | 6 | 116228067 | *GAREM1* | rs81390932 |
| ALGA0036452 | 6 | 116681458 | *-* | rs81390963 |
| DRGA0006717 | 6 | 116701805 | *-* | rs81299090 |
| ALGA0036446 | 6 | 116729394 | *-* | rs81390951 |
| ASGA0093226 | 6 | 117393014 | *ASXL3* | rs81312128 |
| MARC0012805 | 6 | 117440209 | *ASXL3* | rs81280386 |
| ALGA0124272 | 6 | 117505518 | *ASXL3* | rs81304888 |
| ASGA0091558 | 6 | 117519555 | *ASXL3* | rs81475869 |
| WU_10.2_6_109987667 | 6 | 117636766 | *NOL4* | rs341822079 |
| MARC0094627 | 6 | 117716544 | *NOL4* | rs81274928 |
| WU_10.2_6_110172527 | 6 | 117811884 | *NOL4* | rs342920034 |
| CASI0009620 | 6 | 117880883 | *NOL4* | rs325581984 |
| WU_10.2_6_110299648 | 6 | 117938986 | *NOL4* | rs337806275 |
| ALGA0122088 | 6 | 118058538 | *NOL4* | rs81310124 |
| ASGA0105896 | 6 | 118142600 | *NOL4* | rs81306132 |
| MARC0087051 | 6 | 118143865 | *NOL4* | rs81268822 |
| MARC0033633 | 6 | 118154602 | *NOL4* | rs81227635 |
| ASGA0094245 | 6 | 118156567 | *NOL4* | rs81313364 |
| MARC0031537 | 6 | 118367514 | *DTNA* | rs81225967 |
| ASGA0094709 | 6 | 118454924 | *DTNA* | rs81313948 |
| MARC0050983 | 6 | 118547338 | *DTNA* | rs81242840 |
| MARC0073289 | 6 | 118553786 | *DTNA* | rs81259451 |
| DIAS0000385 | 6 | 118615037 | *DTNA* | rs343844669 |
| ALGA0120470 | 6 | 118745133 | *DTNA* | rs81327863 |
| MARC0062521 | 6 | 118794812 | *-* | rs81251085 |
| MARC0070145 | 6 | 118818260 | *MAPRE2* | rs81255529 |
| WU_10.2_6_111134306 | 6 | 118880845 | *MAPRE2* | rs328704161 |
| ASGA0029264 | 6 | 119011410 | *MAPRE2* | rs81391046 |
| MARC0071182 | 6 | 119041972 | *-* | rs81257667 |
| ASGA0029261 | 6 | 119087666 | *ZSCAN30* | rs81391034 |
| ASGA0098709 | 6 | 119223012 | *-* | rs81319025 |
| WU_10.2_6_111760112 | 6 | 119301317 | *GALNT1* | rs341663941 |
| H3GA0055471 | 6 | 119358759 | *GALNT1* | rs81312979 |
| MARC0102757 | 6 | 119382621 | *GALNT1* | rs81279545 |
| MARC0015284 | 6 | 119459403 | *GALNT1* | rs81283256 |
| ALGA0036515 | 6 | 119678992 | *-* | rs81391096 |
| MARC0058155 | 6 | 119738186 | *C18orf21* | rs81247877 |
| MARC0009656 | 6 | 119804586 | *RPRD1A* | rs81258579 |
| ALGA0116229 | 6 | 119953697 | *MOCOS* | rs81345674 |
| MARC0009578 | 6 | 120053311 | *FHOD3* | rs81258152 |
| MARC0042822 | 6 | 120066519 | *FHOD3* | rs81233855 |
| H3GA0018716 | 6 | 120163897 | *FHOD3* | rs81391135 |
| MARC0056265 | 6 | 120185651 | *FHOD3* | rs81246051 |
| ASGA0029292 | 6 | 120247036 | *FHOD3* | rs81391151 |
| MARC0111696 | 6 | 120259899 | *FHOD3* | rs81257158 |
| ALGA0118431 | 6 | 120355907 | *FHOD3* | rs81325333 |
| ASGA0083580 | 6 | 120435160 | *FHOD3* | rs81338371 |
| H3GA0053043 | 6 | 120437893 | *FHOD3* | rs81337502 |
| MARC0087327 | 6 | 120497118 | *FHOD3* | rs81268885 |
| MARC0058096 | 6 | 120511919 | *FHOD3* | rs81247575 |
| ASGA0084114 | 6 | 120532935 | *FHOD3* | rs81476475 |
| ALGA0120843 | 6 | 120571461 | *FHOD3* | rs81476257 |
| MARC0067641 | 6 | 120592135 | *TPGS2* | rs81254792 |
| WU_10.2_6_113488317 | 6 | 120761993 | *KIAA1328* | rs81231720 |
| MARC0109198 | 6 | 120846100 | *KIAA1328* | rs81228069 |
| ALGA0111812 | 6 | 120862959 | *KIAA1328* | rs81476443 |
| ALGA0112500 | 6 | 120954193 | *KIAA1328* | rs81341112 |
| H3GA0056342 | 6 | 121005974 | *KIAA1328* | rs81322965 |
| ALGA0102114 | 6 | 121011821 | *KIAA1328* | rs81476603 |
| MARC0057015 | 6 | 121213301 | *CELF4* | rs81246850 |
| M1GA0008884 | 6 | 121234012 | *CELF4* | rs81391175 |
| ALGA0107778 | 6 | 121319241 | *CELF4* | rs81476356 |
| ASGA0091636 | 6 | 121327915 | *CELF4* | rs81310186 |
| ASGA0094860 | 6 | 121328865 | *CELF4* | rs81314142 |
| MARC0022914 | 6 | 121329970 | *CELF4* | rs81291246 |
| MARC0020625 | 6 | 121373131 | *CELF4* | rs81288778 |
| MARC0093627 | 6 | 121385749 | *CELF4* | rs81272447 |
| MARC0098539 | 6 | 121394974 | *-* | rs81277314 |
| MARC0030522 | 6 | 121498044 | *-* | rs81225276 |
| MARC0086601 | 6 | 121553230 | *-* | rs81269223 |

Supp. Table 2. QTLs, determined by the smoothed FST method (LW_Old vs LW_New)

| ID QTL | SSC | Trait | Class | Group |
| --- | --- | --- | --- | --- |
| 64630 | 5 | Thoracic vertebra number | Conformation | Exterior traits |
| 64681 | 5 | Spinal curvature | Defects | Exterior traits |
| 31802 | 5 | PRRSV susceptibility | Disease susceptibility | Health traits |
| 171144 | 5 | Blood urea level | Blood parameters | Health Traits |
| 65003 | 4 | Heart weight | Anatomy | Meat and carcass trait |
| 125479 | 6 | Intra muscular fat content | Fatness | Meat and carcass trait |
| 153659 | 6 | Ham weight | Anatomy | Meat and carcass trait |
| 153714 | 6 | Loin weight | Anatomy | Meat and carcass trait |
| 160893 | 6 | Conductivity  45minute spost-mortem | Conductivity impedance | Meat and carcass trait |
| 160958 | 6 | Dressing percentage | Anatomy | Meat and carcass trait |
| 167239 | 6 | Backfat at last rib | Fatness | Meat and carcass trait |
| 167248 | 6 | Backfat at tenth rib | Fatness | Meat and carcass trait |
| 23159 | 4 | Shoulder weight | Anatomy | Meat and carcass traits |
| 23166 | 5 | Backfat thickness  Between 3^rd^ and 4^th^ rib | Fatness | Meat and carcass traits |
| 28050 | 4 | Meat colora* | Meat colour | Meat and carcass traits |
| 29605 | 4 | Body weight weaning | Growth | Production traits |
| 66276 | 4 | Bodyweight | Growth | Production traits |
| 18128 | 5 | Number of stillborn | Litter traits | Reproduction traits |
| 18146 | 4 | Gestation length | Reprodictive traits | Reproduction traits |
| 18301 | 6 | Number of mummified pigs | Litter traits | Reproduction traits |
| 31853 | 4 | Corpus luteum number | Litter traits | Reproduction traits |
| 126618 | 5 | Teat number | Reproductive organ | Reproduction traits |

Supp. Table 3. SNPs with the most significant signals, determined by the FLK method (LW_Old vs LW_New)

| Uploaded variation | SSC | Location | Gene | SNP rs |
| --- | --- | --- | --- | --- |
| ALGA0002916 | 1 | 43006479 | *FAM184A* | rs81353735 |
| ASGA0004308 | 1 | 113630812 | *-* | rs81355717 |
| H3GA0003730 | 1 | 221372453 | *DOCK8* | rs81350722 |
| ASGA0000422 | 1 | 3593533 | *-* | rs80804050 |
| WU_10.2_1_4917615 | 1 | 3611749 | *-* | rs80801783 |
| M1GA0000498 | 1 | 3639624 | *-* | rs80867878 |
| H3GA0002682 | 1 | 122909530 | *CEP152* | rs80970028 |
| ALGA0010839 | 1 | 270610430 | *-* | rs81352938 |
| INRA0003613 | 1 | 105918436 | *-* | rs339641713 |
| H3GA0003733 | 1 | 221642261 | *PGM5* | rs80926902 |
| MARC0047721 | 1 | 225077683 | *-* | rs81239814 |
| DRGA0002119 | 1 | 229012388 | *PCSK5* | rs80905481 |
| ALGA0005706 | 1 | 117583391 | *UNC13C* | rs81348106 |
| ALGA0005711 | 1 | 117608243 | *UNC13C* | rs80816756 |
| ALGA0005714 | 1 | 117633924 | *UNC13C* | rs80961292 |
| DRGA0001495 | 1 | 124333001 | *-* | rs81296591 |
| ALGA0005848 | 1 | 124451879 | *-* | rs81348378 |
| ALGA0016905 | 2 | 146761420 | *-* | rs81367186 |
| DRGA0003538 | 2 | 126841331 | *CEP120* | rs81296219 |
| ALGA0011377 | 2 | 3985548 | *-* | rs81357266 |
| WU_10.2_2_148421412 | 2 | 142476638 | *PCDHAC2* | rs320899654 |
| ASGA0012763 | 2 | 147780035 | *-* | rs81367244 |
| H3GA0008207 | 2 | 147728261 | *-* | rs81367259 |
| ALGA0016901 | 2 | 146715517 | *-* | rs81367175 |
| ALGA0120145 | 2 | 147622500 | *-* | rs81327481 |
| ASGA0099180 | 2 | 147654948 | *-* | rs81319612 |
| ASGA0013576 | 3 | 14516653 | *AUTS2* | rs81374217 |
| ASGA0100006 | 3 | 14515470 | *AUTS2* | rs81320648 |
| DBWU0000400 | 3 | 9463123 | *CUX1* | rs55617855 |
| ASGA0013247 | 3 | 9571294 | *CUX1* | rs81370233 |
| INRA0016754 | 4 | 111985089 | *VAV3* | rs328408635 |
| ALGA0028235 | 4 | 111423264 | *FAM102B* | rs80798855 |
| ALGA0026246 | 4 | 82530260 | *-* | rs80851024 |
| ASGA0019027 | 4 | 22504204 | *-* | rs80877304 |
| ALGA0027760 | 4 | 105537200 | *TSPAN2* | rs80792104 |
| INRA0017183 | 4 | 119756297 | *-* | rs327792482 |
| WU_10.2_4_141282346 | 4 | 128834681 | *-* | rs335369842 |
| ASGA0021997 | 4 | 109578232 | *-* | rs80945484 |
| ALGA0024031 | 4 | 22081785 | *-* | rs81381385 |
| ASGA0021815 | 4 | 107101021 | *MAGI3* | rs80855267 |
| WU_10.2_4_117272087 | 4 | 107125678 | *MAGI3* | rs331191282 |
| DRGA0005165 | 4 | 113952913 | *-* | rs81302095 |
| MARC0056159 | 5 | 72681499 | *PDZRN4* | rs80790387 |
| WU_10.2_5_75652966 | 5 | 72710914 | *PDZRN4* | rs343745666 |
| WU_10.2_5_97368087 | 5 | 92707698 | *-* | rs320826225 |
| WU_10.2_5_97383238 | 5 | 92722848 | *-* | rs330643917 |
| ALGA0032748 | 5 | 70121621 | *MICAL3* | rs81385205 |
| WU_10.2_5_75349235 | 5 | 72455402 | *CNTN1* | rs196955486 |
| M1GA0026700 | 5 | 65047600 | *NTF3* | rs81319316 |
| WU_10.2_5_75022148 | 5 | 72169605 | *CNTN1* | rs327321401 |
| WU_10.2_5_4846181 | 5 | 7665916 | *SLC25A17* | rs330340592 |
| H3GA0015326 | 5 | 8052127 | *-* | rs80880715 |
| ASGA0025852 | 5 | 64378343 | *-* | rs81384683 |
| BGIS0001413 | 5 | 38794710 | *GLIPR1* | rs81212454 |
| ALGA0031782 | 5 | 38815027 | *GLIPR1* | rs81383891 |
| WU_10.2_5_75465827 | 5 | 72523648 | *-* | rs327502834 |
| ALGA0033600 | 5 | 92647755 | *-* | rs80985876 |
| M1GA0008007 | 5 | 70222706 | *USP18* | rs81385234 |
| H3GA0016255 | 5 | 38599413 | *CAPS2* | rs81383876 |
| ASGA0025473 | 5 | 38741541 | *-* | rs81383879 |
| ASGA0025477 | 5 | 38772761 | *GLIPR1* | rs80789169 |
| ALGA0030029 | 5 | 6866316 | *MEI1* | rs80990628 |
| MARC0036317 | 5 | 66841468 | *PRMT8* | rs81229819 |
| H3GA0016585 | 5 | 66194434 | *-* | rs80860217 |
| ALGA0032428 | 5 | 65232068 | *-* | rs80828123 |
| MARC0078782 | 5 | 65263327 | *-* | rs80826589 |
| WU_10.2_6_35747679 | 6 | 40449361 | *ZNF536* | rs345502770 |
| ASGA0093426 | 6 | 43809208 | *-* | rs81312339 |
| MARC0072609 | 6 | 44535224 | *ZNF792* | rs81258771 |
| ASGA0102371 | 6 | 78069272 | *-* | rs81323679 |
| MARC0045097 | 6 | 80491095 | *-* | rs81237319 |
| MARC0089589 | 6 | 104183387 | *METTL4* | rs81270901 |
| ASGA0084790 | 6 | 107577819 | *-* | rs81345398 |
| ALGA0113175 | 6 | 107604059 | *-* | rs81341898 |
| ASGA0090610 | 6 | 107705690 | *-* | rs81475846 |
| MARC0020347 | 6 | 107739202 | *-* | rs81289650 |
| ASGA0098807 | 6 | 115348051 | *DSG3* | rs81477249 |
| ASGA0089317 | 6 | 115501657 | *TTR* | rs81475807 |
| MARC0055381 | 6 | 115511066 | *B4GALT6* | rs81245564 |
| DRGA0006714 | 6 | 115826701 | *-* | rs80852301 |
| INRA0022047 | 6 | 115933412 | *RNF138* | rs346414970 |
| ASGA0101965 | 6 | 115989400 | *-* | rs81323160 |
| ALGA0036440 | 6 | 116228067 | *GAREM1* | rs81390932 |
| MARC0012805 | 6 | 117440209 | *ASXL3* | rs81280386 |
| ALGA0124272 | 6 | 117505518 | *ASXL3* | rs81304888 |
| ASGA0091558 | 6 | 117519555 | *ASXL3* | rs81475869 |
| MARC0033633 | 6 | 118154602 | *NOL4* | rs81227635 |
| ASGA0094245 | 6 | 118156567 | *NOL4* | rs81313364 |
| ASGA0094709 | 6 | 118454924 | *DTNA* | rs81313948 |
| ALGA0120470 | 6 | 118745133 | *DTNA* | rs81327863 |
| MARC0070145 | 6 | 118818260 | *MAPRE2* | rs81255529 |
| ASGA0098709 | 6 | 119223012 | *-* | rs81319025 |
| WU_10.2_6_111760112 | 6 | 119301317 | *GALNT1* | rs341663941 |
| MARC0058155 | 6 | 119738186 | *C18orf21* | rs81247877 |
| MARC0009656 | 6 | 119804586 | *RPRD1A* | rs81258579 |
| H3GA0018716 | 6 | 120163897 | *FHOD3* | rs81391135 |
| H3GA0053043 | 6 | 120437893 | *FHOD3* | rs81337502 |
| MARC0087327 | 6 | 120497118 | *FHOD3* | rs81268885 |
| ALGA0112500 | 6 | 120954193 | *KIAA1328* | rs81341112 |
| ASGA0029311 | 6 | 122508849 | *-* | rs81391208 |
| WU_10.2_6_135339230 | 6 | 146934015 | *-* | rs329908175 |
| WU_10.2_6_147223902 | 6 | 159462617 | *ZYG11B* | rs338565316 |
| ALGA0048360 | 8 | 79875615 | *-* | rs81401775 |
| MARC0109216 | 8 | 29664380 | *-* | rs81245798 |
| WU_10.2_8_84148235 | 8 | 79239404 | *IQCM* | rs323143709 |
| WU_10.2_8_31165675 | 8 | 29686508 | *-* | rs337302298 |
| ALGA0048346 | 8 | 79392379 | *IQCM* | rs81401720 |
| ALGA0105578 | 9 | 54113499 | *-* | rs81332927 |
| DRGA0009506 | 9 | 77234719 | *-* | rs81300269 |
| MARC0034655 | 9 | 123180938 | *CACNA1E* | rs81228109 |
| ALGA0055321 | 9 | 126718858 | *-* | rs81417434 |
| ASGA0042615 | 9 | 40290530 | *-* | rs81409416 |
| ALGA0053856 | 9 | 75143144 | *ASB4* | rs81413399 |
| ALGA0117593 | 9 | 9268486 | *XRRA1* | rs81347393 |
| ASGA0094412 | 9 | 9031083 | *CHRDL2* | rs81477194 |
| ASGA0048906 | 10 | 64001935 | *SFMBT2* | rs81427389 |
| ALGA0059100 | 10 | 47515492 | *FRMD4A* | rs81425148 |
| ASGA0046875 | 10 | 17743923 | *KIF26B* | rs81421364 |
| ALGA0059049 | 10 | 47339095 | *-* | rs81425058 |
| WU_10.2_11_3896029 | 11 | 4470233 | *-* | rs319095277 |
| WU_10.2_11_17192379 | 11 | 17087206 | *-* | rs326762479 |
| WU_10.2_11_17102723 | 11 | 16997737 | *-* | rs324807535 |
| ASGA0093177 | 12 | 40379937 | *CCT6B* | rs81312072 |
| WU_10.2_12_41472772 | 12 | 39961733 | *SLFN14* | rs334974208 |
| WU_10.2_12_22810910 | 12 | 22352907 | *PSMD3* | rs327628412 |
| WU_10.2_13_18064705 | 13 | 16415777 | *RBMS3* | rs329196888 |
| ALGA0117303 | 13 | 175911737 | *ROBO1* | rs81347054 |
| ASGA0090020 | 13 | 175912123 | *ROBO1* | rs81308267 |
| WU_10.2_13_186290749 | 13 | 175854183 | *ROBO1* | rs335148968 |
| MARC0066018 | 13 | 61346407 | *ITPR1* | rs81253259 |
| WU_10.2_13_210475313 | 13 | 200399888 | *SIM2* | rs337612047 |
| ASGA0066917 | 14 | 127586033 | *-* | rs80886032 |
| DRGA0014684 | 14 | 127119828 | *SHTN1* | rs80797510 |
| ASGA0060838 | 14 | 6720142 | *SLC39A14* | rs80965810 |
| ALGA0074772 | 14 | 7124862 | *PEBP4* | rs80911839 |
| ASGA0060953 | 14 | 7896731 | *-* | rs80812821 |
| ALGA0075646 | 14 | 16166050 | *-* | rs80938569 |
| WU_10.2_14_8410953 | 14 | 7544973 | *LOXL2* | rs332569839 |
| MARC0036731 | 15 | 77098993 | *GORASP2* | rs81230364 |
| SIRI0001080 | 15 | 1657522 | *-* | rs80919148 |
| WU_10.2_15_147110047 | 15 | 133153146 | *EFHD1* | rs335325248 |
| WU_10.2_15_379190 | 15 | 1244172 | *-* | rs342103080 |
| ALGA0115566 | 16 | 32462883 | *MOCS2* | rs81344847 |
| H3GA0053033 | 16 | 18490090 | *MTMR12* | rs81337389 |
| WU_10.2_16_2024086 | 16 | 1794938 | *-* | rs337674465 |
| ASGA0077857 | 17 | 55108240 | *CYP24A1* | rs80842245 |
| WU_10.2_17_61656964 | 17 | 54928166 | *BCAS1* | rs323332361 |
| WU_10.2_17_61589666 | 17 | 54860869 | *-* | rs323238987 |
| WU_10.2_17_11223820 | 17 | 9860385 | *ZMAT4* | rs333811194 |
| DRGA0016747 | 17 | 41675886 | *-* | rs80933809 |
| MARC0012672 | 17 | 55012137 | *BCAS1* | rs80844442 |
| H3GA0049633 | 17 | 55079324 | *-* | rs80976648 |
| WU_10.2_17_61625706 | 17 | 54896908 | *-* | rs328718092 |
| H3GA0049027 | 17 | 41643251 | *-* | rs80873616 |
| WU_10.2_17_47150684 | 17 | 41659453 | *-* | rs334013074 |
| ALGA0097014 | 18 | 11247266 | *-* | rs81470637 |
| WU_10.2_18_12035129 | 18 | 11343387 | *-* | rs323315349 |
| ASGA0080224 | 18 | 48174205 | *-* | rs81470716 |

Supp. Table 4. QTLs, determined by the FLK method (LW_Old vs LW_New)

| SSC | ID QTL | Trait | Class | Group |
| --- | --- | --- | --- | --- |
| 1 | 29590 | Intramuscular fat content | Texture | Meat and carcass trait |
| 1 | 32101 | Linoleic acid content | Texture | Meat and carcass trait |
| 1 | 32102 | Linoleic acid content | Texture | Meat and carcass trait |
| 1 | 21348 | PRRSV susceptibility | Chimestry | Meat and carcass trait |
| 1 | 65010 | Body weight | Blood parameters | Health Traits |
| 2 | 31840 | Corpus luteum number | Blood parameters | Health Traits |
| 2 | 106216 | Litter size | Texture | Meat and carcass trait |
| 2 | 106221 | Piglet mortality | Chimestry | Meat and carcass trait |
| 2 | 167245 | tenderness score | Texture | Meat and carcass trait |
| 2 | 167246 | Shear force | Texture | Meat and carcass trait |
| 3 | 107258 | Mean corpuscular hemoglobin content | Texture | Meat and carcass trait |
| 3 | 107280 | Mean corpuscular volume | Texture | Meat and carcass trait |
| 4 | 31853 | Corpus luteum number | Blood parameters | Health Traits |
| 4 | 32068 | Palmitic acid content | Chimestry | Meat and carcass trait |
| 4 | 164847 | Conductivity 24 hours postmortem loin | Blood parameters | Health Traits |
| 4 | 66276 | Body weight | Blood parameters | Health Traits |
| 4 | 164848 | Conductivity 24 hours postmortem loin | Blood parameters | Health Traits |
| 4 | 170925 | Total number born alive | Fatty acid content | Meat and carcass trait |
| 5 | 121909 | Backfat between 3rd and 4th last ribs | Fatness | Meat and carcass trait |
| 5 | 167226 | Cooking yield | Blood parameters | Health Traits |
| 5 | 31802 | PRRSV susceptibility | Chimestry | Meat and carcass trait |
| 5 | 64629 | Thoracic vertebra number | Fatty acid content | Meat and carcass trait |
| 5 | 64693 | Thoracolumbar vertebra number | Fatty acid content | Meat and carcass trait |
| 5 | 64681 | Spinal curvature | Flavor | Meat and carcass trait |
| 6 | 153714 | Loin weight | Texture | Meat and carcass trait |
| 6 | 161058 | Conductivity 45 minutes post-mortem | Blood parameters | Health Traits |
| 6 | 161142 | Conductivity 45 minutes post-mortem | Blood parameters | Health Traits |
| 6 | 161032 | Conductivity 45 minutes post-mortem | Blood parameters | Health Traits |
| 6 | 161240 | Conductivity 45 minutes post-mortem | Blood parameters | Health Traits |
| 6 | 160981 | Dressing percentage | Blood parameters | Health Traits |
| 6 | 161184 | Conductivity 45 minutes post-mortem | Blood parameters | Health Traits |
| 6 | 161326 | Conductivity 45 minutes post-mortem | Blood parameters | Health Traits |
| 6 | 160958 | Dressing percentage | Blood parameters | Health Traits |
| 6 | 161222 | Conductivity 45 minutes post-mortem | Blood parameters | Health Traits |
| 6 | 161129 | Conductivity 45 minutes post-mortem | Blood parameters | Health Traits |
| 6 | 161201 | Conductivity 45 minutes post-mortem | Blood parameters | Health Traits |
| 6 | 161075 | Conductivity 45 minutes post-mortem | Blood parameters | Health Traits |
| 6 | 160893 | Conductivity 45 minutes post-mortem | Blood parameters | Health Traits |
| 6 | 161243 | Conductivity 45 minutes post-mortem | Blood parameters | Health Traits |
| 6 | 160925 | Conductivity 45 minutes post-mortem | Blood parameters | Health Traits |
| 6 | 161230 | Conductivity 45 minutes post-mortem | Blood parameters | Health Traits |
| 6 | 161104 | Conductivity 45 minutes post-mortem | Blood parameters | Health Traits |
| 6 | 161105 | Conductivity 45 minutes post-mortem | Blood parameters | Health Traits |
| 6 | 160975 | Conductivity 45 minutes post-mortem | Blood parameters | Health Traits |
| 6 | 160974 | Conductivity 45 minutes post-mortem | Blood parameters | Health Traits |
| 6 | 160972 | Conductivity 45 minutes post-mortem | Blood parameters | Health Traits |
| 6 | 160973 | Conductivity 45 minutes post-mortem | Blood parameters | Health Traits |
| 6 | 161027 | Conductivity 45 minutes post-mortem | Blood parameters | Health Traits |
| 6 | 18119 | Number of stillborn | Chimestry | Meat and carcass trait |
| 6 | 18301 | Number of mummified pigs | Chimestry | Meat and carcass trait |
| 6 | 64724 | Lumbar vertebra number | Texture | Meat and carcass trait |
| 6 | 64728 | Spinal curvature | Flavor | Meat and carcass trait |
| 6 | 65080 | Fat weight total | Fatness | Meat and carcass trait |
| 6 | 66277 | Body weight | Blood parameters | Health Traits |
| 6 | 66298 | Teat number | Flavor | Meat and carcass trait |
| 6 | 121905 | Backfat between 3rd and 4th last ribs | Fatness | Meat and carcass trait |
| 6 | 125479 | Intramuscular fat content | Texture | Meat and carcass trait |
| 6 | 126122 | Conformation score | Blood parameters | Health Traits |
| 6 | 135629 | Feed conversion ratio | Feed intake | Production traits |
| 6 | 140318 | PRRS viral load | Chimestry | Meat and carcass trait |
| 6 | 153659 | Ham weight | Feed intake | Production traits |
| 6 | 160909 | Conductivity 45 minutes post-mortem | Blood parameters | Health Traits |
| 6 | 160911 | Conductivity 45 minutes post-mortem | Blood parameters | Health Traits |
| 6 | 160930 | Conductivity 45 minutes post-mortem | Blood parameters | Health Traits |
| 6 | 160931 | Conductivity 45 minutes post-mortem | Blood parameters | Health Traits |
| 6 | 160932 | Conductivity 45 minutes post-mortem | Blood parameters | Health Traits |
| 6 | 160933 | Conductivity 45 minutes post-mortem | Blood parameters | Health Traits |
| 6 | 160935 | Conductivity 45 minutes post-mortem | Blood parameters | Health Traits |
| 6 | 160950 | Conductivity 45 minutes post-mortem | Blood parameters | Health Traits |
| 6 | 160959 | Conductivity 45 minutes post-mortem | Blood parameters | Health Traits |
| 6 | 160961 | Conductivity 45 minutes post-mortem | Blood parameters | Health Traits |
| 6 | 160962 | Conductivity 45 minutes post-mortem | Blood parameters | Health Traits |
| 6 | 160984 | Conductivity 45 minutes post-mortem | Blood parameters | Health Traits |
| 6 | 160987 | Conductivity 45 minutes post-mortem | Blood parameters | Health Traits |
| 6 | 160994 | Conductivity 45 minutes post-mortem | Blood parameters | Health Traits |
| 6 | 161006 | Conductivity 45 minutes post-mortem | Blood parameters | Health Traits |
| 6 | 161007 | Conductivity 45 minutes post-mortem | Blood parameters | Health Traits |
| 6 | 161017 | Conductivity 45 minutes post-mortem | Blood parameters | Health Traits |
| 6 | 161018 | Conductivity 45 minutes post-mortem | Blood parameters | Health Traits |
| 6 | 161021 | Conductivity 45 minutes post-mortem | Blood parameters | Health Traits |
| 6 | 161029 | Conductivity 45 minutes post-mortem | Blood parameters | Health Traits |
| 6 | 161030 | Conductivity 45 minutes post-mortem | Blood parameters | Health Traits |
| 6 | 161031 | Conductivity 45 minutes post-mortem | Blood parameters | Health Traits |
| 6 | 161040 | Conductivity 45 minutes post-mortem | Blood parameters | Health Traits |
| 6 | 161048 | Conductivity 45 minutes post-mortem | Blood parameters | Health Traits |
| 6 | 161055 | Conductivity 45 minutes post-mortem | Blood parameters | Health Traits |
| 6 | 161059 | Conductivity 45 minutes post-mortem | Blood parameters | Health Traits |
| 6 | 161060 | Conductivity 45 minutes post-mortem | Blood parameters | Health Traits |
| 6 | 161061 | Conductivity 45 minutes post-mortem | Blood parameters | Health Traits |
| 6 | 161071 | Conductivity 45 minutes post-mortem | Blood parameters | Health Traits |
| 6 | 161085 | Conductivity 45 minutes post-mortem | Blood parameters | Health Traits |
| 6 | 161090 | Conductivity 45 minutes post-mortem | Blood parameters | Health Traits |
| 6 | 161107 | Conductivity 45 minutes post-mortem | Blood parameters | Health Traits |
| 6 | 161120 | Conductivity 45 minutes post-mortem | Blood parameters | Health Traits |
| 6 | 161147 | Conductivity 45 minutes post-mortem | Blood parameters | Health Traits |
| 6 | 161150 | Conductivity 45 minutes post-mortem | Blood parameters | Health Traits |
| 6 | 161151 | Conductivity 45 minutes post-mortem | Blood parameters | Health Traits |
| 6 | 161161 | Conductivity 45 minutes post-mortem | Blood parameters | Health Traits |
| 6 | 161174 | Conductivity 45 minutes post-mortem | Blood parameters | Health Traits |
| 6 | 161175 | Conductivity 45 minutes post-mortem | Blood parameters | Health Traits |
| 6 | 161176 | Conductivity 45 minutes post-mortem | Blood parameters | Health Traits |
| 6 | 161177 | Conductivity 45 minutes post-mortem | Blood parameters | Health Traits |
| 6 | 161179 | Conductivity 45 minutes post-mortem | Blood parameters | Health Traits |
| 6 | 161187 | Conductivity 45 minutes post-mortem | Blood parameters | Health Traits |
| 6 | 161195 | Conductivity 45 minutes post-mortem | Blood parameters | Health Traits |
| 6 | 161219 | Conductivity 45 minutes post-mortem | Blood parameters | Health Traits |
| 6 | 161232 | Conductivity 45 minutes post-mortem | Blood parameters | Health Traits |
| 6 | 161235 | Conductivity 45 minutes post-mortem | Blood parameters | Health Traits |
| 6 | 161244 | Conductivity 45 minutes post-mortem | Blood parameters | Health Traits |
| 6 | 167239 | Backfat at last rib | Fatness | Meat and carcass trait |
| 6 | 167240 | Backfat at last rib | Fatness | Meat and carcass trait |
| 6 | 167248 | Backfat at tenth rib | Fatness | Meat and carcass trait |
| 8 | 22138 | Mean corpuscular hemoglobin concentration | Texture | Meat and carcass trait |
| 8 | 22141 | Mean corpuscular volume | Texture | Meat and carcass trait |
| 8 | 22142 | Mean corpuscular volume | Texture | Meat and carcass trait |
| 8 | 22143 | Mean corpuscular volume | Texture | Meat and carcass trait |
| 9 | 22995 | Body weight birth | Blood parameters | Health Traits |
| 9 | 23172 | Shoulder weight | Flavor | Meat and carcass trait |
| 9 | 29538 | Average daily gain | Growth | Production traits |
| 9 | 65105 | Lean meat weight | Texture | Meat and carcass trait |
| 9 | 66258 | Daily feed intake | Blood parameters | Health Traits |
| 9 | 125483 | Intramuscular fat content | Texture | Meat and carcass trait |
| 10 | 31880 | Corpus luteum number | Blood parameters | Health Traits |
| 10 | 107273 | Mean corpuscular hemoglobin concentration | Texture | Meat and carcass trait |
| 11 | 55880 | Cryptorchidism | Blood parameters | Health Traits |
| 11 | 55881 | Cryptorchidism | Blood parameters | Health Traits |
| 11 | 65112 | Fat percentage in carcass | Blood parameters | Health Traits |
| 11 | 167224 | Dressing percentage | Blood parameters | Health Traits |
| 11 | 65108 | Fat percentage in carcass | Blood parameters | Health Traits |
| 11 | 65109 | Lean meat percentage | Texture | Meat and carcass trait |
| 11 | 65110 | Fat weight total | Fatness | Meat and carcass trait |
| 12 | 121898 | Meat color b* | Texture | Meat and carcass trait |
| 13 | 18134 | Number of stillborn | Chimestry | Meat and carcass trait |
| 14 | 23006 | Body weight birth | Blood parameters | Health Traits |
| 14 | 29574 | Average backfat thickness | Fatness | Meat and carcass trait |
| 14 | 32128 | Linoleic acid content | Texture | Meat and carcass trait |
| 14 | 140331 | PRRS viral load | Chimestry | Meat and carcass trait |
| 14 | 171152 | Mean corpuscular hemoglobin content | Texture | Meat and carcass trait |
| 14 | 178883 | Number of mummified pigs | Chimestry | Meat and carcass trait |
| 14 | 178884 | Number of mummified pigs | Chimestry | Meat and carcass trait |
| 14 | 178885 | Number of mummified pigs | Chimestry | Meat and carcass trait |
| 14 | 179299 | Litter weight total | Texture | Meat and carcass trait |
| 15 | 15128 | Bilirubin level | Blood parameters | Health Traits |
| 15 | 170629 | Average backfat thickness | Fatness | Meat and carcass trait |
| 15 | 170646 | Lean meat percentage | Texture | Meat and carcass trait |
| 15 | 170898 | Days to 100 kg | Blood parameters | Health Traits |
| 15 | 167231 | muscle protein percentage | Chimestry | Meat and carcass trait |
| 15 | 167228 | pH 24 hr post-mortem loin | Chimestry | Meat and carcass trait |
| 15 | 167229 | Cooking yield | Blood parameters | Health Traits |
| 15 | 22961 | Litter weight total | Texture | Meat and carcass trait |
| 15 | 167230 | Drip loss | Blood parameters | Health Traits |
| 15 | 95254 | Meat color b* | Texture | Meat and carcass trait |
| 15 | 95256 | Meat color a* | Texture | Meat and carcass trait |
| 15 | 95263 | PH for Longissmus dorsi | Chimestry | Meat and carcass trait |
| 15 | 95264 | Meat color b* | Texture | Meat and carcass trait |
| 15 | 95265 | Meat color a* | Texture | Meat and carcass trait |
| 15 | 95259 | Meat color b* | Texture | Meat and carcass trait |
| 15 | 95262 | pH 24 hr post-mortem loin | Chimestry | Meat and carcass trait |
| 15 | 95266 | Meat color b* | Texture | Meat and carcass trait |
| 15 | 95267 | pH 24 hr post-mortem loin | Chimestry | Meat and carcass trait |
| 15 | 95268 | PH for Longissmus dorsi | Chimestry | Meat and carcass trait |
| 15 | 95269 | Meat color a* | Texture | Meat and carcass trait |
| 15 | 95270 | Meat color b* | Texture | Meat and carcass trait |
| 15 | 95271 | Meat color a* | Texture | Meat and carcass trait |
| 15 | 95272 | PH for Longissmus dorsi | Chimestry | Meat and carcass trait |
| 15 | 95273 | Meat color b* | Texture | Meat and carcass trait |
| 15 | 95274 | Meat color b* | Texture | Meat and carcass trait |
| 15 | 95275 | PH for Longissmus dorsi | Chimestry | Meat and carcass trait |
| 15 | 95276 | Meat color a* | Texture | Meat and carcass trait |
| 15 | 95277 | PH for Longissmus dorsi | Chimestry | Meat and carcass trait |
| 15 | 95278 | PH for Longissmus dorsi | Chimestry | Meat and carcass trait |
| 15 | 95279 | PH for Longissmus dorsi | Chimestry | Meat and carcass trait |
| 15 | 95280 | PH for Longissmus dorsi | Chimestry | Meat and carcass trait |
| 15 | 95281 | Meat color a* | Texture | Meat and carcass trait |
| 15 | 95298 | Testicular length | Fatty acid content | Meat and carcass trait |
| 15 | 95339 | Testicular percentage | Fatty acid content | Meat and carcass trait |
| 15 | 167232 | Shear force | Texture | Meat and carcass trait |
| 15 | 167234 | Juiciness score | Texture | Meat and carcass trait |
| 15 | 167235 | tenderness score | Texture | Meat and carcass trait |
| 16 | 64664 | Lumbar vertebra number | Texture | Meat and carcass trait |
| 16 | 95388 | Arachidic acid content | Fatty acid content | Meat and carcass trait |
| 16 | 164852 | pH 24 hr post-mortem loin | Chimestry | Meat and carcass trait |
| 17 | 23157 | Backfat thickness between 3rd and 4th rib | Fatness | Meat and carcass trait |
| 17 | 23164 | Backfat thickness between 3rd and 4th rib | Fatness | Meat and carcass trait |
| 17 | 164855 | pH 24 hr post mortem ham | Chimestry | Meat and carcass trait |
| 18 | 64671 | Lumbar vertebra number | Texture | Meat and carcass trait |
| 18 | 126136 | Conformation score | Blood parameters | Health Traits |

Supp. Table 5. SNPs with the most significant signals, determined by the smoothed FST method (LW_1 vs LW_New)

| Uploaded variation | SSC | Location | Gene | SNP rs |
| --- | --- | --- | --- | --- |
| INRA0043941 | 14 | 45509383 | TTC28 | rs343327654 |
| ALGA0077373 | 14 | 45556841 | TTC28 | rs80978873 |
| ASGA0063205 | 14 | 45602780 | TTC28 | rs80969675 |
| ASGA0063206 | 14 | 45643020 | TTC28 | rs80811002 |
| ASGA0063207 | 14 | 45656863 | TTC28 | rs80954165 |
| DRGA0013807 | 14 | 45727087 | TTC28 | rs80971789 |
| MARC0092574 | 14 | 45766858 | TTC28 | rs80882617 |
| MARC0047822 | 14 | 45827810 | TTC28 | rs80968146 |
| ALGA0077380 | 14 | 45859200 | TTC28 | rs80806354 |
| ALGA0077382 | 14 | 46031357 | - | rs80824834 |
| H3GA0040075 | 14 | 46084074 | - | rs80993233 |
| H3GA0040076 | 14 | 46148505 | ZNRF3 | rs80832470 |
| ALGA0077388 | 14 | 46219359 | ZNRF3 | rs80883638 |
| MARC0005951 | 14 | 46297598 | KREMEN1 | rs80920419 |
| ASGA0063217 | 14 | 46323552 | KREMEN1 | rs80928428 |
| INRA0043964 | 14 | 46418356 | EWSR1 | rs325891452 |
| BGIS0000437 | 14 | 46427612 | EWSR1 | rs80995717 |
| DBNP0002145 | 14 | 46436960 | EWSR1 | rs45435082 |
| ALGA0077394 | 14 | 46473900 | AP1B1 | rs80851887 |
| ALGA0077399 | 14 | 46494819 | AP1B1 | rs80784711 |
| MARC0048650 | 14 | 46520497 | AP1B1 | rs80960734 |
| ALGA0077404 | 14 | 46563973 | NEFH | rs80809319 |
| MARC0008762 | 14 | 46640387 | THOC5 | rs80963093 |
| MARC0036724 | 14 | 46738288 | - | rs80796509 |

Supp. Table 6. QTLs, determined by the smoothed FST method (LW_1 vs LW_New)

| ID QTL | SSC | Trait | Class | Group |
| --- | --- | --- | --- | --- |
| 16007 | 14 | Ratio of lifetime nonproductive days to herd life | Lifetime production | Production traits |
| 23007 | 14 | Body weight birth | Growth | Production traits |
| 23008 | 14 | Body weight birth | Growth | Production traits |
| 107339 | 14 | White blood cell numbe | Imune capacity | Health traits |
| 178883 | 14 | Mummified pigs | Litter traits | Reproduction traits |
| 178884 | 14 | Mummified pigs | Litter traits | Reproduction traits |
| 178885 | 14 | Mummified pigs | Litter traits | Reproduction traits |
| 178893 | 14 | Mummified pigs | Litter traits | Reproduction traits |
| 178894 | 14 | Mummified pigs | Litter traits | Reproduction traits |
| 178895 | 14 | Mummified pigs | Litter traits | Reproduction traits |
| 178896 | 14 | Mummified pigs | Litter traits | Reproduction traits |
| 178897 | 14 | Mummified pigs | Litter traits | Reproduction traits |
| 178898 | 14 | Mummified pigs | Litter traits | Reproduction traits |
| 178899 | 14 | Mummified pigs | Litter traits | Reproduction traits |
| 178900 | 14 | Mummified pigs | Litter traits | Reproduction traits |
| 178901 | 14 | Mummified pigs | Litter traits | Reproduction traits |
| 179293 | 14 | Litter size | Litter traits | Reproduction traits |
| 179294 | 14 | Litter size | Litter traits | Reproduction traits |
| 179299 | 14 | Litter weight total | Litter traits | Reproduction traits |
| 179305 | 14 | Litter size | Litter traits | Reproduction traits |
| 179306 | 14 | Litter size | Litter traits | Reproduction traits |

Supp. Table 7. SNPs with the most significant signals, determined by the smoothed FST method (LW_2 vs LW_New)

| Uploaded variation | SSC | Location | Gene | SNP rs |
| --- | --- | --- | --- | --- |
| ASGA0003188 | 1 | 62307146 | - | rs81354504 |
| ALGA0003898 | 1 | 62318714 | - | rs81354508 |
| DRGA0001068 | 1 | 62371709 | - | rs81296935 |
| INRA0002659 | 1 | 62534115 | - | rs346300123 |
| ALGA0003900 | 1 | 62583607 | - | rs80977812 |
| ASGA0003191 | 1 | 62598591 | - | rs80996124 |
| DRGA0001072 | 1 | 62645599 | - | rs80813230 |
| ALGA0003901 | 1 | 62681226 | - | rs80808398 |
| DRGA0001073 | 1 | 62759784 | - | rs80854829 |
| INRA0002667 | 1 | 62781138 | - | rs328370740 |
| ASGA0003194 | 1 | 62811729 | - | rs80868692 |
| ASGA0097561 | 1 | 62926723 | MANEA | rs80890495 |
| ALGA0003904 | 1 | 62986317 | - | rs80910541 |
| H3GA0001876 | 1 | 63012075 | - | rs81354534 |
| ASGA0003200 | 1 | 63104570 | - | rs81354550 |
| ASGA0091357 | 1 | 63189393 | - | rs81309827 |
| MARC0112869 | 1 | 63225607 | - | rs81286784 |
| MARC0059892 | 1 | 63244752 | - | rs81249333 |
| CASI0007774 | 1 | 63262614 | - | rs318827837 |
| ALGA0003917 | 1 | 63282337 | - | rs81354560 |
| M1GA0000987 | 1 | 63294966 | - | rs81354561 |
| H3GA0001881 | 1 | 63316569 | - | rs81354562 |
| ALGA0003918 | 1 | 63431796 | - | rs80791165 |
| INRA0002686 | 1 | 63513495 | - | rs319749497 |
| ASGA0003205 | 1 | 63566792 | - | rs80870997 |
| MARC0024388 | 1 | 63704733 | UFL1 | rs80930000 |
| DRGA0001092 | 1 | 63726056 | FHL5 | rs80921830 |
| DRGA0001093 | 1 | 63753893 | FHL5 | rs80855411 |
| ALGA0102804 | 1 | 63773754 | - | rs80870517 |
| ALGA0124376 | 1 | 63800886 | - | rs80878579 |
| ALGA0003927 | 1 | 63856265 | - | rs80867872 |
| MARC0059407 | 1 | 63930516 | GPR63 | rs80976465 |
| ALGA0003935 | 1 | 63996109 | - | rs80997522 |
| ASGA0003216 | 1 | 64079226 | KLHL32 | rs80918268 |
| DRGA0001098 | 1 | 64119625 | KLHL32 | rs80978351 |
| ASGA0003219 | 1 | 64135700 | KLHL32 | rs80958906 |
| ASGA0003223 | 1 | 64154811 | KLHL32 | rs80791584 |
| MARC0104726 | 1 | 64203044 | KLHL32 | rs80846286 |
| ASGA0003229 | 1 | 64301394 | MMS22L | rs81354571 |
| H3GA0001888 | 1 | 64395202 | MMS22L | rs81354573 |
| ALGA0003946 | 1 | 64452788 | MMS22L | rs80798219 |
| DRGA0001102 | 1 | 64553243 | - | rs80868763 |
| ALGA0003951 | 1 | 64579689 | - | rs80958033 |
| ASGA0003233 | 1 | 64591470 | - | rs80952489 |
| ALGA0003952 | 1 | 64614854 | - | rs80995170 |
| ALGA0003953 | 1 | 64674794 | - | rs80996334 |
| H3GA0001895 | 1 | 64745294 | - | rs80929393 |
| H3GA0001896 | 1 | 64877553 | - | rs80794355 |
| ALGA0003960 | 1 | 64908479 | - | rs80864801 |
| MARC0095827 | 1 | 64945916 | U6 | rs80905307 |
| ASGA0003235 | 1 | 64978117 | - | rs80788846 |
| WU_10.2_1_73702285 | 1 | 65152428 | - | rs325200009 |
| ALGA0003968 | 1 | 65337586 | - | rs81354584 |
| ALGA0003972 | 1 | 65373610 | - | rs80893006 |
| DRGA0001106 | 1 | 65460886 | U6 | rs80886843 |
| ASGA0003239 | 1 | 65474177 | - | rs80979692 |
| INRA0002726 | 1 | 65537992 | - | rs341934411 |
| ASGA0003244 | 1 | 65649768 | - | rs80891284 |
| MARC0075306 | 1 | 65711728 | - | rs80838693 |
| ALGA0003988 | 1 | 65759072 | - | rs80872224 |
| INRA0002742 | 1 | 65796820 | - | rs334645174 |
| ALGA0003991 | 1 | 65814755 | - | rs80946739 |
| ALGA0003992 | 1 | 65841441 | POU3F2 | rs80950025 |
| ALGA0003995 | 1 | 65859940 | - | rs80857181 |
| DIAS0002827 | 1 | 65875605 | FBXL4 | rs80924453 |
| ALGA0004000 | 1 | 65918133 | FBXL4 | rs80892026 |
| ALGA0004002 | 1 | 65953598 | - | rs80915364 |
| ASGA0003261 | 1 | 66008742 | - | rs80792488 |
| ALGA0004005 | 1 | 66034686 | - | rs80882527 |
| ALGA0004006 | 1 | 66047984 | - | rs80821723 |

Supp. Table 8. QTLs, determined by the smoothed FST method (LW_2 vs LW_New)

| ID QTL | SSC | Trait | Class | Group |
| --- | --- | --- | --- | --- |
| 18270 | 1 | Mummified pigs | Litter traits | Reproduction traits |
| 30983 | 1 | Maternal infanticide | Behavioral | Exterior traits |
| 32098 | 1 | Palmitic acid content | Fatty acid content | Meat and carcass trait |
| 95694 | 1 | Ham weight | Anatomy | Meat and carcass trait |
| 126113 | 1 | Top line conformation | Conformation | Exterior traits |

Supp. Table 9. SNPs with the most significant signals, determined by the smoothed FST method (LW_3 vs LW_New)

| Uploaded variation | SSC | Location | Gene | SNP rs |
| --- | --- | --- | --- | --- |
| MARC0021796 | 15 | 77070266 | GORASP2 | rs81291638 |
| MARC0036731 | 15 | 77098993 | GORASP2 | rs81230364 |
| H3GA0044547 | 15 | 77128890 | GORASP2 | rs80837643 |
| MARC0046707 | 15 | 77157077 | - | rs80844498 |
| ALGA0085987 | 15 | 77178701 | TLK1 | rs80965573 |
| H3GA0044550 | 15 | 77232829 | TLK1 | rs80852223 |
| ALGA0085989 | 15 | 77318065 | TLK1 | rs80999734 |
| MARC0098380 | 15 | 77364176 | - | rs80799468 |
| ALGA0085996 | 15 | 77440817 | - | rs80840446 |
| ASGA0069907 | 15 | 77469771 | - | rs80981676 |
| ASGA0069910 | 15 | 77529731 | METTL8 | rs80889278 |
| H3GA0044561 | 15 | 77542253 | METTL8 | rs80993791 |
| H3GA0044562 | 15 | 77566996 | METTL8 | rs81000506 |
| ALGA0119841 | 6 | 130871443 | - | rs81327100 |
| MARC0028469 | 6 | 130937009 | - | rs81222864 |
| WU_10.2_6_121362776 | 6 | 130969638 | - | rs321468972 |
| SIRI0001502 | 6 | 131101648 | - | rs321214830 |
| ALGA0036715 | 6 | 131175703 | - | rs81391518 |
| ALGA0036714 | 6 | 131202858 | - | rs81391515 |
| MARC0071162 | 6 | 131244998 | - | rs81257397 |
| ALGA0036723 | 6 | 131278864 | - | rs81391530 |
| ALGA0036718 | 6 | 131300502 | - | rs81391526 |
| H3GA0018809 | 6 | 131381466 | - | rs81391555 |
| INRA0022280 | 6 | 131404149 | - | rs338651373 |
| ALGA0036729 | 6 | 131459409 | - | rs81391549 |
| ASGA0029413 | 6 | 131514715 | - | rs81391537 |
| ASGA0029412 | 6 | 131528172 | - | rs80996593 |
| ALGA0115834 | 6 | 131595818 | - | rs81345174 |
| MARC0063627 | 6 | 131659278 | - | rs81252273 |
| ALGA0106465 | 6 | 131681990 | - | rs81333964 |
| ASGA0098556 | 6 | 131692557 | - | rs81476067 |
| ALGA0116715 | 6 | 131715732 | - | rs81476575 |
| MARC0009864 | 6 | 131767738 | ADGRL2 | rs81258912 |
| WU_10.2_6_122065838 | 6 | 131840097 | ADGRL2 | rs340095665 |
| DIAS0000786 | 6 | 131919402 | ADGRL2 | rs328311629 |
| ALGA0036742 | 6 | 132005399 | ADGRL2 | rs81391579 |
| WU_10.2_6_122438275 | 6 | 132210828 | ADGRL2 | rs322523427 |
| WU_10.2_6_122469298 | 6 | 132241849 | ADGRL2 | rs196952550 |
| MARC0076441 | 6 | 132363552 | ADGRL2 | rs81261721 |
| MARC0094511 | 6 | 132429025 | - | rs81274905 |
| DRGA0017543 | 6 | 132432954 | - | rs339759836 |
| MARC0046692 | 6 | 132501629 | - | rs81237150 |
| WU_10.2_6_122896738 | 6 | 132592988 | - | rs328085218 |
| H3GA0018816 | 6 | 132710655 | - | rs81391595 |
| H3GA0018818 | 6 | 132846185 | - | rs81391604 |
| H3GA0018820 | 6 | 132875916 | - | rs81391613 |
| ASGA0029430 | 6 | 132919641 | - | rs81391623 |
| WU_10.2_6_123307483 | 6 | 132999380 | - | rs332101662 |
| WU_10.2_6_123338444 | 6 | 133030115 | - | rs335597078 |
| DRGA0006798 | 6 | 133134545 | - | rs81299123 |
| ALGA0036763 | 6 | 133162201 | - | rs81391647 |
| H3GA0018823 | 6 | 133185154 | - | rs81391651 |
| ASGA0029438 | 6 | 133219375 | - | rs81391658 |
| MARC0046041 | 6 | 133376793 | - | rs81237495 |
| INRA0022295 | 6 | 133437673 | - | rs335277441 |
| H3GA0018827 | 6 | 133453545 | - | rs81391706 |
| ALGA0036794 | 6 | 133500969 | - | rs81391712 |

Supp. Table 10. QTLs, determined by the smoothed FST method (LW_3 vs LW_New)

| ID QTL | SSC | Trait | Class | Group |
| --- | --- | --- | --- | --- |
| 15128 | 15 | Maternal infanticide | Behavioral | Exterior traits |
| 22961 | 15 | Bilirubin level | Blood parameters | Health Traits |
| 30994 | 15 | Average backfat thickness | Fatness | Meat and carcass trait |
| 153659 | 6 | Backfat at last lumbar | Fatness | Meat and carcass trait |
| 153714 | 6 | Backfat at last rib | Fatness | Meat and carcass trait |
| 160893 | 6 | Backfat at last rib | Fatness | Meat and carcass trait |
| 160909 | 6 | Backfat at last rib | Fatness | Meat and carcass trait |
| 160911 | 6 | Backfat at last rib | Fatness | Meat and carcass trait |
| 160925 | 6 | Backfat at tenth rib | Fatness | Meat and carcass trait |
| 160930 | 6 | Backfat at tenth rib | Fatness | Meat and carcass trait |
| 160931 | 6 | Backfat at tenth rib | Fatness | Meat and carcass trait |
| 160932 | 6 | Backfat at tenth rib | Fatness | Meat and carcass trait |
| 160933 | 6 | Conductivity 45 minutes post-mortem | Conductivity impedance | Meat and carcass trait |
| 160935 | 6 | Conductivity 45 minutes post-mortem | Conductivity impedance | Meat and carcass trait |
| 160950 | 6 | Conductivity 45 minutes post-mortem | Conductivity impedance | Meat and carcass trait |
| 160958 | 6 | Conductivity 45 minutes post-mortem | Conductivity impedance | Meat and carcass trait |
| 160959 | 6 | Conductivity 45 minutes post-mortem | Conductivity impedance | Meat and carcass trait |
| 160961 | 6 | Conductivity 45 minutes post-mortem | Conductivity impedance | Meat and carcass trait |
| 160962 | 6 | Conductivity 45 minutes post-mortem | Conductivity impedance | Meat and carcass trait |
| 160972 | 6 | Conductivity 45 minutes post-mortem | Conductivity impedance | Meat and carcass trait |
| 160973 | 6 | Conductivity 45 minutes post-mortem | Conductivity impedance | Meat and carcass trait |
| 160974 | 6 | Conductivity 45 minutes post-mortem | Conductivity impedance | Meat and carcass trait |
| 160975 | 6 | Conductivity 45 minutes post-mortem | Conductivity impedance | Meat and carcass trait |
| 160981 | 6 | Conductivity 45 minutes post-mortem | Conductivity impedance | Meat and carcass trait |
| 160984 | 6 | Conductivity 45 minutes post-mortem | Conductivity impedance | Meat and carcass trait |
| 160987 | 6 | Conductivity 45 minutes post-mortem | Conductivity impedance | Meat and carcass trait |
| 160994 | 6 | Conductivity 45 minutes post-mortem | Conductivity impedance | Meat and carcass trait |
| 161006 | 6 | Conductivity 45 minutes post-mortem | Conductivity impedance | Meat and carcass trait |
| 161007 | 6 | Conductivity 45 minutes post-mortem | Conductivity impedance | Meat and carcass trait |
| 161017 | 6 | Conductivity 45 minutes post-mortem | Conductivity impedance | Meat and carcass trait |
| 161018 | 6 | Conductivity 45 minutes post-mortem | Conductivity impedance | Meat and carcass trait |
| 161021 | 6 | Conductivity 45 minutes post-mortem | Conductivity impedance | Meat and carcass trait |
| 161027 | 6 | Conductivity 45 minutes post-mortem | Conductivity impedance | Meat and carcass trait |
| 161029 | 6 | Conductivity 45 minutes post-mortem | Conductivity impedance | Meat and carcass trait |
| 161030 | 6 | Conductivity 45 minutes post-mortem | Conductivity impedance | Meat and carcass trait |
| 161031 | 6 | Conductivity 45 minutes post-mortem | Conductivity impedance | Meat and carcass trait |
| 161032 | 6 | Conductivity 45 minutes post-mortem | Conductivity impedance | Meat and carcass trait |
| 161040 | 6 | Conductivity 45 minutes post-mortem | Conductivity impedance | Meat and carcass trait |
| 161048 | 6 | Conductivity 45 minutes post-mortem | Conductivity impedance | Meat and carcass trait |
| 161055 | 6 | Conductivity 45 minutes post-mortem | Conductivity impedance | Meat and carcass trait |
| 161058 | 6 | Conductivity 45 minutes post-mortem | Conductivity impedance | Meat and carcass trait |
| 161059 | 6 | Conductivity 45 minutes post-mortem | Conductivity impedance | Meat and carcass trait |
| 161060 | 6 | Conductivity 45 minutes post-mortem | Conductivity impedance | Meat and carcass trait |
| 161061 | 6 | Conductivity 45 minutes post-mortem | Conductivity impedance | Meat and carcass trait |
| 161071 | 6 | Conductivity 45 minutes post-mortem | Conductivity impedance | Meat and carcass trait |
| 161075 | 6 | Conductivity 45 minutes post-mortem | Conductivity impedance | Meat and carcass trait |
| 161085 | 6 | Conductivity 45 minutes post-mortem | Conductivity impedance | Meat and carcass trait |
| 161090 | 6 | Conductivity 45 minutes post-mortem | Conductivity impedance | Meat and carcass trait |
| 161104 | 6 | Conductivity 45 minutes post-mortem | Conductivity impedance | Meat and carcass trait |
| 161105 | 6 | Conductivity 45 minutes post-mortem | Conductivity impedance | Meat and carcass trait |
| 161107 | 6 | Conductivity 45 minutes post-mortem | Conductivity impedance | Meat and carcass trait |
| 161120 | 6 | Conductivity 45 minutes post-mortem | Conductivity impedance | Meat and carcass trait |
| 161129 | 6 | Conductivity 45 minutes post-mortem | Conductivity impedance | Meat and carcass trait |
| 161142 | 6 | Conductivity 45 minutes post-mortem | Conductivity impedance | Meat and carcass trait |
| 161147 | 6 | Conductivity 45 minutes post-mortem | Conductivity impedance | Meat and carcass trait |
| 161150 | 6 | Conductivity 45 minutes post-mortem | Conductivity impedance | Meat and carcass trait |
| 161151 | 6 | Conductivity 45 minutes post-mortem | Conductivity impedance | Meat and carcass trait |
| 161161 | 6 | Conductivity 45 minutes post-mortem | Conductivity impedance | Meat and carcass trait |
| 161174 | 6 | Conductivity 45 minutes post-mortem | Conductivity impedance | Meat and carcass trait |
| 161175 | 6 | Conductivity 45 minutes post-mortem | Conductivity impedance | Meat and carcass trait |
| 161176 | 6 | Conductivity 45 minutes post-mortem | Conductivity impedance | Meat and carcass trait |
| 161177 | 6 | Conductivity 45 minutes post-mortem | Conductivity impedance | Meat and carcass trait |
| 161179 | 6 | Conductivity 45 minutes post-mortem | Conductivity impedance | Meat and carcass trait |
| 161184 | 6 | Conductivity 45 minutes post-mortem | Conductivity impedance | Meat and carcass trait |
| 161187 | 6 | Conductivity 45 minutes post-mortem | Conductivity impedance | Meat and carcass trait |
| 161195 | 6 | Conductivity 45 minutes post-mortem | Conductivity impedance | Meat and carcass trait |
| 161201 | 6 | Conductivity 45 minutes post-mortem | Conductivity impedance | Meat and carcass trait |
| 161219 | 6 | Conductivity 45 minutes post-mortem | Conductivity impedance | Meat and carcass trait |
| 161222 | 6 | Conductivity 45 minutes post-mortem | Conductivity impedance | Meat and carcass trait |
| 161230 | 6 | Conductivity 45 minutes post-mortem | Conductivity impedance | Meat and carcass trait |
| 161232 | 6 | Conductivity 45 minutes post-mortem | Conductivity impedance | Meat and carcass trait |
| 161235 | 6 | Conductivity 45 minutes post-mortem | Conductivity impedance | Meat and carcass trait |
| 161240 | 6 | Conductivity 45 minutes post-mortem | Conductivity impedance | Meat and carcass trait |
| 161243 | 6 | Conductivity 45 minutes post-mortem | Conductivity impedance | Meat and carcass trait |
| 161244 | 6 | Conductivity 45 minutes post-mortem | Conductivity impedance | Meat and carcass trait |
| 167231 | 6 | Conductivity 45 minutes post-mortem | Conductivity impedance | Meat and carcass trait |
| 167236 | 6 | Conductivity 45 minutes post-mortem | Conductivity impedance | Meat and carcass trait |
| 167238 | 6 | Conductivity 45 minutes post-mortem | Conductivity impedance | Meat and carcass trait |
| 167239 | 6 | Conductivity 45 minutes post-mortem | Conductivity impedance | Meat and carcass trait |
| 167240 | 6 | Conductivity 45 minutes post-mortem | Conductivity impedance | Meat and carcass trait |
| 167241 | 6 | Dressing percentage | Anatomy | Meat and carcass trait |
| 167243 | 6 | Dressing percentage | Anatomy | Meat and carcass trait |
| 167248 | 6 | Ham weight | Anatomy | Meat and carcass trait |
| 167250 | 6 | Lean meat percentage | Fatness | Meat and carcass trait |
| 167257 | 6 | Loin weight | Anatomy | Meat and carcass trait |
| 167258 | 6 | Loin weight | Anatomy | Meat and carcass trait |
| 170629 | 15 | Muscle protein percentage | Chimestry | Meat and carcass trait |
| 170646 | 15 | Days to 100 kg | Growth | Production traits |
| 170898 | 15 | Litter weight total | Litter traits | Reproduction traits |

Supp. Table 11. SNPs with the most significant signals, determined by the smoothed FST method (LW_4 vs LW_New)

| Uploaded variation | SSC | Location | Gene | SNP rs |
| --- | --- | --- | --- | --- |
| ASGA0023017 | 4 | 123920076 | CCDC18 | rs80790526 |
| ASGA0023013 | 4 | 123966445 | CCDC18 | rs80914682 |
| WU_10.2_4_135566902 | 4 | 124012054 | TMED5 | rs326091598 |
| ALGA0029233 | 4 | 124037786 | - | rs80921658 |
| ALGA0029232 | 4 | 124048185 | MTF2 | rs80871521 |
| H3GA0014723 | 4 | 124089737 | MTF2 | rs80990190 |
| MARC0056797 | 4 | 124163620 | - | rs80865148 |
| ASGA0023001 | 4 | 124186629 | - | rs80947379 |
| WU_10.2_4_135844283 | 4 | 124216278 | U6 | rs342988395 |
| WU_10.2_4_135906408 | 4 | 124228412 | DIPK1A | rs321429919 |
| ALGA0029201 | 4 | 124398947 | EVI5 | rs80968273 |
| H3GA0014703 | 4 | 124421379 | EVI5 | rs80835478 |
| H3GA0014699 | 4 | 124464881 | EVI5 | rs80930480 |
| H3GA0014695 | 4 | 124482750 | EVI5 | rs80928870 |
| WU_10.2_4_136122846 | 4 | 124499977 | EVI5 | rs80893135 |
| WU_10.2_4_136181224 | 4 | 124567768 | EVI5 | rs318521790 |
| ALGA0029186 | 4 | 124598790 | EVI5 | rs80978362 |
| WU_10.2_4_136246749 | 4 | 124618201 | GFI1 | rs328627001 |
| H3GA0014684 | 4 | 124664729 | - | rs81380724 |
| WU_10.2_4_136353777 | 4 | 124684774 | - | rs328269066 |
| ASGA0022968 | 4 | 124706568 | RPAP2 | rs81380721 |
| WU_10.2_4_136460091 | 4 | 124791451 | GLMN | rs322700348 |
| WU_10.2_4_136522900 | 4 | 125061511 | BRDT | rs332945547 |
| WU_10.2_4_136543493 | 4 | 125079457 | - | rs328328631 |
| ASGA0027714 | 6 | 19730662 | KIFC3 | rs81391898 |
| ALGA0034691 | 6 | 19760890 | - | rs81391786 |
| WU_10.2_6_17831619 | 6 | 19779760 | - | rs81299289 |
| MARC0065967 | 6 | 19810566 | - | rs81252955 |
| DIAS0000453 | 6 | 19869309 | CFAP20 | rs334905777 |
| ALGA0034684 | 6 | 19903679 | CFAP20 | rs81391709 |
| ALGA0104811 | 6 | 19926572 | CFAP20 | rs81332032 |
| M1GA0025169 | 6 | 19932639 | CFAP20 | rs81346829 |
| H3GA0054827 | 6 | 19940940 | CFAP20 | rs81475784 |
| DIAS0002910 | 6 | 19956188 | CFAP20 | rs81218446 |
| WU_10.2_6_17996915 | 6 | 19973006 | CSNK2A2 | rs345477428 |
| WU_10.2_6_18061416 | 6 | 20003167 | CSNK2A2 | rs330893487 |
| ALGA0115587 | 6 | 20040749 | - | rs81344881 |
| ALGA0105770 | 9 | 74428112 | SGCE | rs81333124 |
| ASGA0043786 | 9 | 74614086 | - | rs81413366 |
| DRGA0009479 | 9 | 74725542 | PPP1R9A | rs81300512 |
| DRGA0009480 | 9 | 74746108 | PPP1R9A | rs81299849 |
| ALGA0053850 | 9 | 74785106 | PPP1R9A | rs80904264 |
| ASGA0043791 | 9 | 75000208 | PON3 | rs81413387 |
| CASI0008905 | 9 | 75039565 | PON2 | rs333740492 |
| ASGA0043792 | 9 | 75087018 | ASB4 | rs81413393 |
| DRGA0009485 | 9 | 75128908 | ASB4 | rs81300125 |
| ALGA0053856 | 9 | 75143144 | ASB4 | rs81413399 |
| ALGA0053857 | 9 | 75177787 | ASB4 | rs81413401 |
| ASGA0043796 | 9 | 75199308 | ASB4 | rs81413407 |
| H3GA0027806 | 9 | 75223028 | - | rs81413410 |
| ASGA0043798 | 9 | 75259764 | PDK4 | rs81413411 |
| MARC0053461 | 9 | 75290228 | U6 | rs81243664 |
| CASI0008397 | 9 | 75314108 | ASB4 | rs331524345 |
| DRGA0009488 | 9 | 75332568 | DYNC1I1 | rs81300327 |
| ASGA0043805 | 9 | 75407604 | DYNC1I1 | rs81413421 |
| ASGA0043811 | 9 | 75469583 | DYNC1I1 | rs81413429 |
| ASGA0043814 | 9 | 75490123 | DYNC1I1 | rs81413432 |
| WU_10.2_9_82963397 | 9 | 75584709 | DYNC1I1 | rs339756139 |
| ALGA0124187 | 9 | 75718984 | DYNC1I1 | rs81304306 |
| ASGA0089825 | 9 | 75723619 | DYNC1I1 | rs81308004 |
| WU_10.2_9_83271497 | 9 | 75858422 | SLC25A13 | rs320992982 |
| ALGA0053884 | 9 | 75989142 | SLC25A13 | rs81413453 |
| ASGA0043822 | 9 | 76052645 | SLC25A13 | rs81413460 |
| ASGA0095468 | 9 | 76081740 | SLC25A13 | rs81477200 |
| ASGA0043825 | 9 | 76087912 | SLC25A13 | rs81413464 |
| WU_10.2_9_83556488 | 9 | 76105135 | SLC25A13 | rs340241892 |
| ALGA0053892 | 9 | 76182853 | SLC25A13 | rs81413466 |
| ALGA0053893 | 9 | 76220300 | SLC25A13 | rs81413467 |
| DRGA0009493 | 9 | 76248997 | SLC25A13 | rs336498572 |

\

Supp. Table 12. QTLs, determined by the smoothed FST method (LW_4 vs LW_New)

| ID QTL | SSC | Trait | Class | Group |
| --- | --- | --- | --- | --- |
| 31853 | 4 | Corpus luteum number | Litter traits | Reproduction traits |
| 37455 | 9 | Teat number | Reproductive organ | Reproduction traits |
| 125483 | 9 | Intramuscular fat content | Fatness | Meat and carcass trait |
| 161058 | 6 | Conductivity 45 minutes post-mortem | Conductivity impedance | Meat and carcass trait |
