# Supplementary figures and images for: Genome-wide analysis of genetic diversity and artificial selection in Large White pigs in Russia
Source: PeerJ. 2021 Jul 2;9:e11595. doi: 10.7717/peerj.11595 (PMC8256806; doi:10.7717/peerj.11595)

D

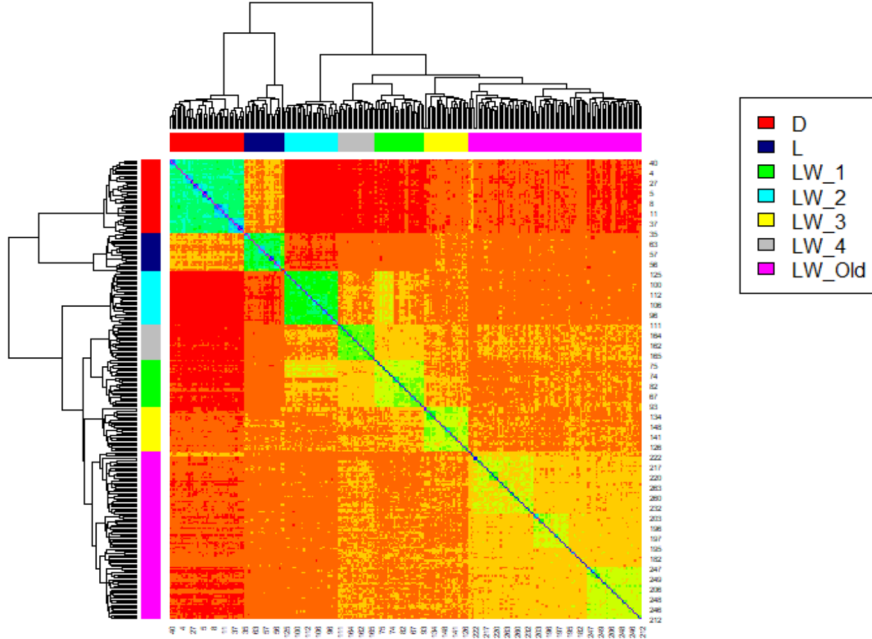

Supplement: Supplemental Information 5 — This is the high resolution version of the Fig. 3D showing the heatmap of the samples. [file peerj-09-11595-s005.pdf]
